# Sugar, amino acid and inorganic ion profiling of the honeydew from different hemipteran species feeding on *Abies alba* and *Picea abies*

**DOI:** 10.1371/journal.pone.0228171

**Published:** 2020-01-24

**Authors:** Basel Shaaban, Victoria Seeburger, Annette Schroeder, Gertrud Lohaus

**Affiliations:** 1 Molecular Plant Science / Plant Biochemistry, University of Wuppertal, Wuppertal, Germany; 2 Apicultural State Institute, University of Hohenheim, Stuttgart, Germany; Natural Resources Canada, CANADA

## Abstract

Several hemipteran species feed on the phloem sap of plants and produce large amounts of honeydew that is collected by bees to produce honeydew honey. Therefore, it is important to know whether it is predominantly the hemipteran species or the host plant to influence the honeydew composition. This is particularly relevant for those botanical and zoological species from which the majority of honeydew honey originates. To investigate this issue, honeydew from two *Cinara* species located on *Abies alba* as well as from two *Cinara* and two *Physokermes* species located on *Picea abies* were collected. Phloem exudates of the host plants were also analyzed. Honeydew of all species contained different proportions of hexoses, sucrose, melezitose, erlose, and further di- and trisaccharides, whereas the phloem exudates of the host trees contained no trisaccharides. Moreover, the proportions of sugars differed significantly between hemipteran species feeding on the same tree species. Sucrose hydrolysis and oligosaccharide formation was shown in whole-body homogenates of aphids. The type of the produced oligosaccharides in the aphid-extracts correlated with the oligosaccharide composition in the honeydew of the different aphid species. The total contents of amino acids and inorganic ions in the honeydew were much lower than the sugar content. Glutamine and glutamate were predominant amino acids in the honeydew of all six hemipteran species and also in the phloem exudates of both tree species. Potassium was the dominant inorganic ion in all honeydew samples and also in the phloem exudate. Statistical analyses reveal that the sugar composition of honeydew is determined more by the hemipteran species than by the host plant. Consequently, it can be assumed that the sugar composition of honeydew honey is also more influenced by the hemipteran species than by the host tree.

## Introduction

Many insects of the order Hemiptera, including most aphids and coccids, feed on the phloem sap of their respective host plants [1]. Phloem sap is generally dominated by sucrose, with concentrations ranging from 0.7 to 1.5 M [2,3,4]. Some plant species also translocate oligosaccharides of the raffinose family, such as members of the Oleaceae, or sugar alcohols, such as members of the Rosaceae, in addition to sucrose [5,6].

Phloem sap also contains amino acids with a concentration of 50 to 200 mM. In several plant species, especially GLU, GLN, ASP, and ASN are dominant amino acids [3,4,7,8]. For several insects, nine amino acids are essential (HIS, ILE, LEU, LYS, MET, PHE, THR, TRP, VAL) [1]. Although all amino acids were found in the phloem sap [3,9], it is not an ideal diet for insects because of its high osmotic pressure, the low ratio of amino acids compared to sugars, and the ratio of essential-to-non-essential amino acid, which is lower in phloem sap than in the insect protein [1]. Therefore, phloem feeders ingest phloem sugars in quantities exceeding their carbon requirement to fulfil their metabolic need, and high concentrations of sugars in modified composition are egested as honeydew [10,11].

Honeydew is mainly composed of sugars, but it also contains inorganic ions, amino acids, proteins and other compounds [12,13,14,15]. The honeydew composition varies between different insect species; it can also be influenced by different host plant species, seasonal changes or different environmental conditions [9,16,17]. In addition, the honeydew composition may be influenced by variation in ant-aphid interaction [18]. Moreover, there is a considerable variation in the composition within samples from a particular aphid species [18].

Different monosaccharides (glucose, fructose), disaccharides (e.g. sucrose, trehalose, maltose) and trisaccharides (e.g. melezitose, erlose, raffinose) were found in honeydew [15,17,19,20] but not in the phloem sap of several tree species, where only sucrose was present [2,21]. In willow trees, for example, sucrose was the only sugar found in the phloem sap, whereas the honeydew of a phloem feeding willow aphid (*Tuberolachnus salignus*) contained different mono-, di-, and trisaccharides [21]. Moreover, large variations of the sugar composition in honeydew of three aphid species feeding on the same tree species (*Populus tremula*) were observed [18].

For the osmotic regulation of the phloem feeding insects, it is important that they form oligosaccharides from the sucrose ingested with the phloem sap [10,22,23]. A positive relationship between the dietary sucrose concentration and the oligosaccharide content in the honeydew was shown for different aphid species [10,22,23]. The oligosaccharides are probably synthesized by several enzymes in the gut of the aphids [10,24,25].

Associated bacteria may also be involved in the nutrient metabolism in the insects [1]. One of the best-studied symbioses of this type are the symbioses of *Buchnera* and aphids, where the microorganisms are involved in the provision of essential amino acids for aphids [26,27,28,29]. In the case of oligosaccharides, the aphids rather than the associated microbiota mediate the synthesis of these sugars [10].

When floral nectar is scare, bees often collect honeydew that has fallen onto plants. Therefore, it is important for the honey production industry to know its composition. The production of large amounts of honeydew is known for insects feeding on conifers, i.e. fir, spruce, or pine and also on deciduous trees, such as oak or lime [30]. Honeydew honey is also called forest, spruce or fir honey. Forest honey contains more di- and oligosaccharides than flower honey [31]. Nottbohm and Lucius [32] found melezitose in honeydew honey, of which Hudson and Sherwood [33] already knew that it was responsible for the crystallization of honey in the combs. This can have negative effects on the honey production process. There are some, mainly older, publications about the proportion of melezitose in honeydew of hemipteran feeding aphids on conifers, but the data are partly inconsistent [34]. Liebig [20] reports a share of 15% melezitose of the total sugar content in honeydew of *Cinara pectinatae* feeding on *Abies alba*, whereas in other studies, no melezitose was detected [35]. The quality of honey depends, in addition to several other factors, also on the quantity of melezitose and other oligosaccharides. Therefore, it is important to know which insect species or plant species could be responsible for high melezitose contents in honeydew. This is particularly important for hemipteran species and conifer species which are associated with honeydew honey.

The aim of this study was to determine the proportions of melezitose and other sugars, as well as amino acids and further ions in the honeydew of different hemipteran species in order to examine whether the honeydew compositions differ among hemipteran species and/or among conifer species. Therefore, we collected honeydew droplets directly from two Coccidae species (*Physokermes piceae* and *Physokermes hemicryphus*) and from two Lachninae species (*Cinara pilicornis* and *Cinara piceae*) located on spruce (*Picea abies* (L.) H. Kast.) and also from two Lachninae species (*Cinara pectinatae* and *Cinara confinis*) located on fir (*Abies alba* Mill.). The analyzed hemipteran species are important producers of honeydew on conifers in Germany and other countries of Central Europe [30]. We determined the sugars, amino acids, and inorganic ions in the honeydew and examined the formation of oligosaccharides in different *Cinara* species. The results were compared to the corresponding proportions of the different compounds in phloem exudates of the tree species.

## Material and methods

### Plant species, hemipteran species, and collection of honeydew

The honeydew and plant samples were collected in five spruce (*Picea abies*) or fir (*Abies alba*) stands of Baden-Wuerttemberg (Germany). The geographic coordinates for the stands are 48°66’41”N, 8°32’54”E; 48°48’19”N, 8°37’13”E; 48°31’56”N, 8°47’04”E; 48°95’58”N, 8°70’60”E; and 49°00’40”N, 10°07’07”E. Honeydew of *Cinara pectinatae* (Nördlinger, 1880) and *Cinara confinis* (Koch, 1856) located on *A*. *alba* (Mill.) and of *Cinara pilicornis* (Hartig, 1841), *Cinara piceae* (Panzer, 1801), *Physokermes piceae* (Schrank, 1801) and *Physokermes hemicryphus* (Dalman, 1826) located on *P*. *abies* ((L.) H. Kast.) was collected. The taller trees were about 20–30 years old. Honeydew was collected from the lower or overhanging branches. Field experiments were carried out from May to July 2016 and also 2017. To minimize diurnal influences, all samples were collected at mid-day (between 11 am and 3 pm). For each species, at least 15 honeydew samples were collected from 15 colonies feeding on different tree individuals. The honeydew from different individuals in one colony was directly collected with micropipettes and pooled up to a volume of at least 1 μL per sample. All droplets were still liquid, their age, meaning the time of release by the insect, however, was unknown. The samples were collected in plastic tubes, immediately frozen and stored at -80°C until analysis.

### Collection of phloem exudates

Phloem exudate of bark from *A*. *alba* and *P*. *abies* was collected parallel to the honeydew sampling from May to July 2016 and also 2017 at mid-day (between 11 am and 3 pm). For each tree species, six samples of phloem exudate were prepared. According to the method of Hijaz and Killiny [36], roughly 2 cm long pieces of bark were placed in a 0.5 ml plastic tube with a small hole at its bottom. This tube was placed inside a 2 ml plastic tube. The samples were then centrifuged at 5000 rpm for 20 min at 4°C. This exudate consists mainly of phloem sap, but there may also be small amounts of xylem and other cell sap from the wound surface during the cutting process. The samples were stored -80°C until analysis.

### Ethics statement

As per the authors’ institutions’ guidelines as well as applicable national regulations, no ethics approval was required or obtained for the present study. This study was carried out in spruce (*P*. *abies)* or fir (*A*. *alba)* stands in Baden-Wuerttemberg (Germany). No specific permissions were required for these locations. We collected honeydew from aphids and scale insects, as well as material of *P*. *abies* and *A*. *alba*. Neither the insects nor the plants are protected by German law and no endangered or protected species were involved in this study.

### HPLC analyses of sugars, amino acids, and inorganic ions

Sugars, amino acids, and inorganic ions were analyzed via different HPLC systems. Honeydew and phloem exudates were measured directly. Sugar standards (0–500 μM), amino acid standards (0–20 μM), and standards for inorganic ions (0–1000 μM) were measured in parallel. A calibration curve was created for each sugar, amino acid, or inorganic ion. The peak areas in the measured chromatograms were evaluated with an integration program (Chromeleon 7.2, Dionex Corp, Sunnyvale, CA, United States). The concentrations of sugars, amino acids, or inorganic ions were determined with the help of the calibration curves for each of the different substances. In order to make the results of the different hemipteran species or biological origin (honeydew or phloem exudate) comparable, the proportion of each sugar, amino acid or ions of the total sugar, as well as the amino acid or ion concentration was calculated.

#### Sugar analyses

The sugars in honeydew and bark exudates were analyzed according to Lohaus and Schwerdtfeger [8]. Therefore, an anion exchange column and pulse amperometric detection were used. Standards (glucose, fructose, sucrose, trehalose, melibiose, maltose, isomaltose, maltulose, isomaltulose, melezitose, erlose, raffinose, 1-kestose, isomaltotriose, maltotriose, nidrose, stachyose) were measured in parallel. The identification of each sugar was based on the comparison of the retention time of the different peaks with that of the standards. Furthermore, the obtained results were checked regularly with the standard addition method. The co-elution of sugars in the samples with known standards confirmed our assumption. Long-chain oligosaccharides (degree of polymerization (DP) ≥ 5) were analyzed with the same system, with the difference that the anion exchange column was eluted isocratically with 200 mM NaOH instead of 80 mM NaOH [37]. For the non-availability of higher oligosaccharide standards, long-chain oligosaccharides were quantified against verbascose (DP5) standard and presented as verbascose equivalents.

#### Amino acid analyses

The analysis of free amino acids was performed according to Göttlinger et al. [38]. Amino acids with a primary amino group were processed by precolumn derivatization with o-phtaldialdehyde, amino acids with a secondary amino group (e.g. proline) with fluorenylmethyloxycarbonyl. The derivates were detected by fluorescence.

#### Analyses of inorganic ions

Anions and cations were analyzed separately according to Göttlinger et al. [38]. The ions were detected by their electronic conductivity.

### Enzyme activity in whole-body homogenates of aphids

The enzyme activities were analyzed in *C*. *pectinatae* feeding on *A*. *alba* and *C*. *pilicornis* feeding on *P*. *abies*. About five *C*. *pectinatae-*individuals and about ten *C*. *pilicornis-*individuals, were collected from the host plant using a paintbrush. After collection, they were homogenized in a cooled plastic tube with the help of a pestle. 300 μL sucrose solution (10%, pH 7) were added to 20 mg aphid homogenate and incubated at 30°C. After 0, 30, 60, and 120 minutes, aliquots (50 μL) were taken and 50 μL NaOH (200 mM) were added to inactivate any enzymes or microbe-activity. The solution was then centrifuged (13,000 *xg*, 30 sec) and the supernatants were taken for sugar analysis by HPLC (as described above). The experiment was performed at least three times for each aphid species.

### Statistical analyses

Data for sugar, amino acids, or ion proportions in honeydew are shown as means (± SD). The means of each of the sugars, amino acids, or ions in the honeydew of the six hemipteran species were compared separately to check for significant differences. Skewness and kurtosis were calculated to capture the distribution of the dataset; normal distribution was assumed if skewness values were less than 2 and kurtosis values were less than 7 [39]. Moreover, Levene’s tests were applied to test for homogeneity of variances for the data of each metabolite or ion. When data conformed to the normality assumption but failed on homogeneity of variances, analysis of variance was performed using the Welch’s test followed by post-hoc test (Games-Howell test). If both normality and homogeneity assumptions were confirmed, a one-way ANOVA was performed. Subsequently, post-hoc tests (Tukey’s HSD) were carried out (p-value ≤ 0.05).

For the enzyme activities in the *Cinara* species a nonparametric test (Mann Whitney U) was carried out to test for significant differences in the mean values.

To conduct a non-metric multidimensional scaling (NMDS) for sugar, amino acid, and ion proportions of honeydew samples for all hemipteran species, data were reconstructed into similarity matrices using the Euclidean distance [40]. They were analyzed with the help of NMDS ordiplots. The fit of the ordination compared to the original sample ranking was assessed using the stress function. The ordination represents the data when the stress value is less than 0.2 [41]. The analysis was performed using the ‘Vegan’ package with the *metaMDS* routine of the program “R” [42].

Permutational Multivariate Analysis of Variance (PERMANOVA, [43]) was performed to identify the relative importance of the variables hemipteran species and tree species on the honeydew composition. Furthermore, Permutational Analysis of Multivariate Dispersions (PERMDISP, [44]) was performed to test the homogeneity of multivariate dispersions and to distinguish between location and dispersion effects in case of significant PERMANOVA values. Both analyses are based on Euclidean distance measures, they were performed using the ‘Vegan’ package with the *betadisper* routine for PERMDISP and *adonis* routine for PERMANOVA of the program “R”. For the PERMANOVA, 999 permutations were applied.

All statistical analyses were performed using R (version 3.5.1, www.r-project.org) and SPSS (version 24.0, IBM, Cooperation).

## Results

### Sugars, amino acids, and inorganic ions in honeydew

**Fig 1:**
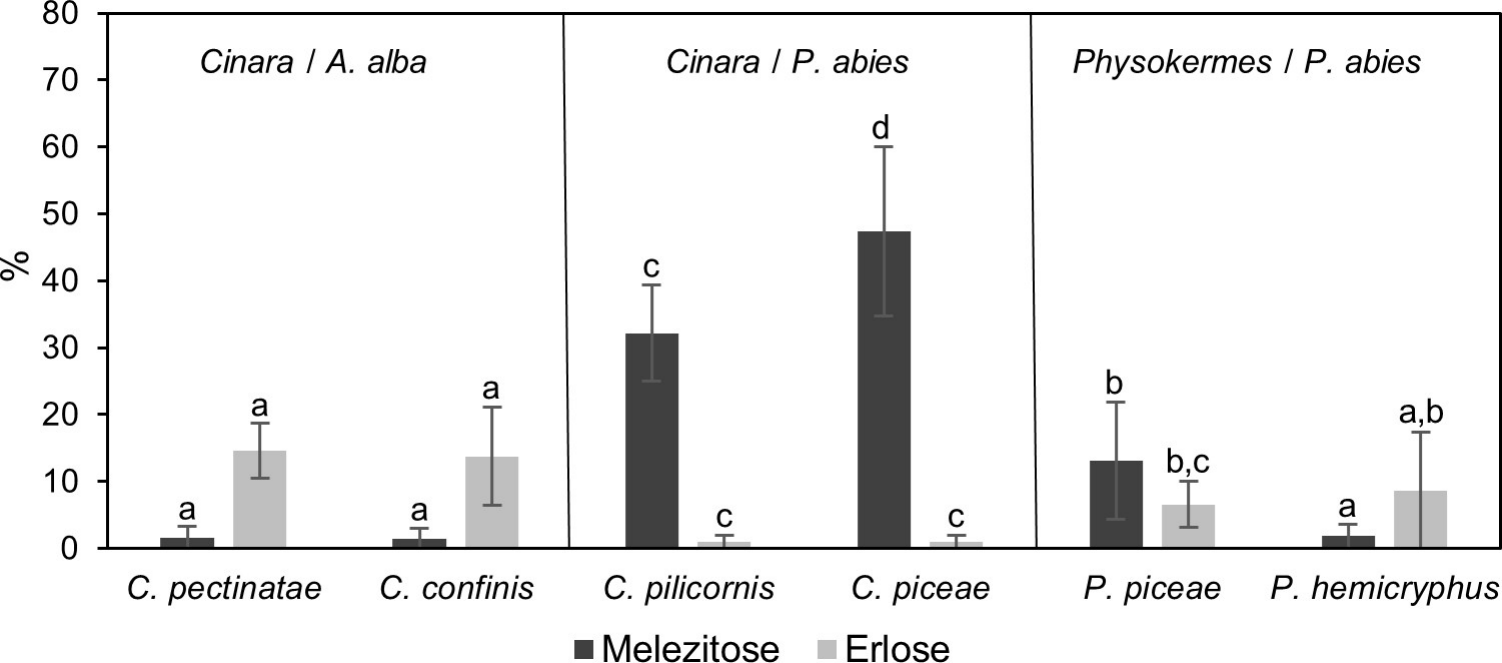
Melezitose and erlose proportion in the honeydew of six hemipteran species feeding on *A*. *alba* and *P*. *abies*. All values are mean proportions (%) of n = 15 independent measurements ± SD. Data were taken from Table 1. Different letters represent significant differences in melezitose and erlose proportion between different hemipteran species (Tukey’s HSD; *p* < 0.05).

Honeydew, produced by *C*. *pectinatae* and *C*. *confinis* on *A*. *alba* and by *C*. *piceae*, *C*. *pilicornis*, *P*. *piceae* and *P*. *hemicryphus* located on *P*. *abies* were analyzed for sugars, amino acids, and inorganic ions. The monosaccharides glucose and fructose, as well as the disaccharide sucrose, were found in all samples (Table 1). In general, the proportion of fructose was higher than the proportion of glucose. The trisaccharides, melezitose and erlose represented also major components of honeydew. There were, however, significant differences between the hemipteran species (Fig 1). Honeydew, produced by *C*. *piceae* located on spruce, revealed the highest proportion of melezitose (mean ± SD; 48 ± 13%), followed by *C*. *pilicornis* (mean ± SD; 36 ± 8%), whereas honeydew produced by *P*. *piceae* and *P*. *hemicryphus* located on spruce and by the Lachninae species *C*. *pectinatae* and *C*. *confinis* on fir showed much lower proportions of melezitose. A reverse picture emerges when analyzing the proportion of erlose: high proportions of erlose were found in honeydew of *C*. *pectinatae* and *C*. *confinis*, medium in *P*. *piceae* and *P*. *hemicryphus*, and low in *C*. *pilicornis* and *C*. *piceae* (Fig 1).

**Table 1:** Sugar composition of the honeydew of six hemipteran species feeding on *Abies alba* or *Picea abies*.

	*Abies alba*		*Picea abies*			
Sugar [%]	*Cinara pectinatae*	*Cinara confinis*	*Cinara pilicornis*	*Cinara piceae*	*Physokermes piceae*	*Physokermes hemicryphus*
Glucose (glu)	12 ± 8^a^	6 ± 6^a,b^	7 ± 10^a,b^	4 ± 3^b^	10 ± 4^a,b^	14 ± 10^a^
Fructose (fru)	13 ± 4^a^	49 ± 12^d^	25 ± 6^b,c^	34 ± 8^c^	30 ± 10^c^	19 ± 12^a,b^
Sucrose (suc)	51 ± 13^a^	24 ± 16^b,c^	30 ± 13^b^	11 ± 8^c^	35 ± 16^a,b^	48 ± 22^a^
Trehalose (tre)	2 ± 2^a,b^	2 ± 1^a,b^	0 ± 0^a^	2 ± 1^b^	3 ± 2^b^	2 ± 3^b^
Maltose (mal)	0 ± 0^a^	2 ± 3^a,b^	1 ± 2^a^	0 ± 0^a^	0 ± 0^a^	4 ± 4^b^
Further disaccharides (fds)	0 ± 0^a,b^	1 ± 1^b^	0 ± 0^a^	0 ± 0^a^	0 ± 0^a,b^	0 ± 0^a,b^
Melezitose (mel)	2 ± 2^a^	2 ± 2^a^	36 ± 8^c^	48 ± 13^d^	13 ± 9^b^	2 ± 5^a^
Erlose (erl)	15 ± 4^a^	14 ± 7^a^	1 ± 2^c^	1 ± 2^c^	7 ± 4^b,c^	9 ± 10^a,b^
1-Kestose* (kes)	4 ± 5^a^	0 ± 0^b^	0 ± 0^b^	0 ± 0^b^	1 ± 2^b^	2 ± 2^a,b^
Further trisaccharides (fts)	1 ± 1^a^	0 ± 0^b^	0 ± 0^b^	0 ± 0^b^	0 ± 0^b^	0 ± 0^b^
Further oligosaccharides	≤ 1	≤ 1	≤ 1	≤ 1	≤ 1	≤ 1

All values are mean proportions (%) of n = 15 independent measurements ± SD.

Different letters represent significant differences between the sugar proportion in honeydew of the different hemipteran species.

Further disaccharides: isomaltose, isomaltulose, maltulose, melibiose, and turanose.

Further trisaccharides: isomaltotriose, maltotriose, and raffinose.

*peak of kestose was not completely separated from nigerose and stachyose peak.

Further oligosaccharides: oligosaccharides with a degree of polymerization ≥ 4.

The disaccharides trehalose and maltose occurred in minor proportions in most honeydew samples (Table 1). Several honeydew samples of *C*. *pectinatae*, *P*. *piceae* and *P*. *hemicryphus* also showed a minor peak in the chromatogram with the retention time of 1-kestose. However, this peak could also represent nigerose and stachyose, because they could not be separated in the chromatogram. Melibiose, isomaltose, turanose, maltulose, isomaltulose, maltotriose, isomaltotriose, raffinose were found not at all or only in single samples, but these sugars never constituted more than 1–2% of the total sugar content. The proportions of long-chain oligosaccharides (DP ≥ 5) were below 1% in all honeydew samples.

Despite the very low amino acids concentration in honeydew, all proteinogenic amino acids and some further amino compounds could be detected (Table 2). The main amino acids were GLN, GLU, PRO, and the essential amino acid HIS. The proportions of the other essential amino acids were low. No significant differences could be detected for most of the amino acids in the honeydew of the different hemipteran species,

**Table 2:** Amino acid composition of the honeydew of six hemipteran species feeding on *Abies alba* or *Picea abies*.

	*Abies alba*		*Picea abies*			
Amino acid [%]	*Cinara pectinatae*	*Cinara confinis*	*Cinara pilicornis*	*Cinara piceae*	*Physokermes piceae*	*Physokermes hemicryphus*
Glutamate (GLU)	16 ± 17^a^	6 ± 7^a^	17 ± 17^a^	15 ± 15^a^	7 ± 8^a^	10 ± 16^a^
Glutamine (GLN)	23 ± 21^a^	23 ± 30^a^	26 ± 29^a^	17 ± 21^a^	37 ± 26^a^	17 ± 21^a^
Aspartate (ASP)	15 ± 15^a^	4 ± 4^b^	7 ± 6^b^	3 ± 2^b^	3 ± 5^b^	9 ± 12^b^
Asparagine (ASN)	10 ± 11^a^	3 ± 3^a^	3 ± 4^a^	3 ± 4^a^	4 ± 5^a^	7 ± 12^a^
Proline (PRO)	10 ± 6^a^	20 ± 25^a^	8 ± 12^a^	6 ± 8^a^	5 ± 6^a^	14 ± 12^a^
Glycine (GLY)	6 ± 9^a^	1 ± 2^a^	5 ± 8^a^	8 ± 15^a^	12 ± 14^a^	5 ± 12^a^
Serine (SER)	4 ± 3^a^	3 ± 3^a^	4 ± 5^a^	8 ± 12^a^	4 ± 8^a^	2 ± 4^a^
Alanine (ALA)	6 ± 11^a^	2 ± 4^a,b^	4 ± 6^a,b^	6 ± 5^b^	5 ± 4^a,b^	2 ± 3^a,b^
Tyrosine (TYR)	0 ± 1^a^	0 ± 1^a^	3 ± 4^a,b^	2 ± 2^a,b^	4 ± 6^b^	2 ± 4^a,b^
Arginine (ARG)	1 ± 4^a^	1 ± 1^a^	1 ± 2^a^	2 ± 3^a^	1 ± 1^a^	0 ± 0^a^
Histidine (HIS)	4 ± 5^a^	27 ± 26^b^	5 ± 6^a^	14 ± 19^a^	4 ± 5^a^	20 ± 21^a^
Lysine (LYS)	2 ± 6^a^	0 ± 1^a^	1 ± 2^a^	5 ± 13^a^	0 ± 1^a^	3 ± 10^a^
Threonine (THR)	2 ± 6^a^	1 ± 2^a^	2 ± 4^a^	2 ± 4^a^	2 ± 4^a^	1 ± 2^a^
Valine (VAL)	1 ± 1^a^	2 ± 4^a^	3 ± 3^a^	3 ± 3^a^	1 ± 2^a^	1 ± 2^a^
Isoleucine (ILE)	0 ± 1^a^	1 ± 1^a,b^	1 ± 1^a,b^	1 ± 1^b^	1 ± 1^a,b^	0 ± 1^a,b^
Leucine (LEU)	0 ± 0^a^	1 ± 1^a,b^	2 ± 4^b^	1 ± 1^a,b^	1 ± 1^a,b^	1 ± 1^a,b^
Phenylalanine (PHE)	0 ± 0^a^	2 ± 3^b^	1 ± 1^a^	1 ± 2^a^	1 ± 2^a,b^	0 ± 1^a^
Tryptophan (TRP)	0 ± 0^a^	0 ± 1^a^	2 ± 3^a^	1 ± 2^a^	1 ± 2^a^	1 ± 2^a^
Methionine (MET)	0 ± 0^a^	0 ± 0^a^	0 ± 0^a^	0 ± 0^a^	0 ± 0^a^	0 ± 0^a^
Non-proteinogenic amino acids (NP)	4 ± 9^a^	3 ± 1^a^	3 ± 1^a^	3 ± 1^a^	6 ± 1^a^	3 ± 1^a^

All values are mean proportions (%) of n = 15 independent measurements ± SD.

Different letters represent significant differences between the amino acid proportion in honeydew of the different hemipteran species.

Non-proteinogenic amino acids: ß-alanine, γ-amino-butyric acid, homoserine, phosphoserine, ornithine, and taurine.

The main anions in honeydew were phosphate (PO_4_^3-^) and chloride (Cl^-^), and potassium (K^+)^ was the most abundant cation (Table 3). Significant differences between the hemipteran species were mainly detected for chloride, phosphate, and ammonium (NH_4_^+^). The proportion of phosphate was particularly high in the honeydew of *Physokermes* species.

**Table 3:** Inorganic cation and anion composition of the honeydew of six hemipteran species feeding on *Abies alba* or *Picea abies*.

	*Abies alba*		*Picea abies*			
Ion [%]	*Cinara pectinatae*	*Cinara confinis*	*Cinara pilicornis*	*Cinara piceae*	*Physokermes piceae*	*Physokermes hemicryphus*
**Cations**						
Potassium (K^+^)	49 ± 10^a^	81 ± 10^b^	72 ± 18^c^	69 ± 15^d^	77 ± 14^e^	82 ± 8^f^
Sodium (Na^+^)	9 ± 11^a,b^	10 ± 7^a,b^	8 ± 6^b^	2 ± 1^a^	2 ± 1^a^	7 ± 8^a,b^
Ammonium (NH_4_^+^)	35 ± 29^a^	2 ± 3^c^	12 ± 16^b,c^	24 ± 15^a,b^	16 ± 15^a,b^	5 ± 5^c^
Magnesium (Mg^2+^)	2 ± 1^a^	4 ± 1^a^	4 ± 3^a^	2 ± 2^a^	3 ± 2^a^	5 ± 4^a^
Calcium (Ca^2+^)	2 ± 1^a,b^	2 ± 1^a,b^	4 ± 4^b^	3 ± 3^a,b^	1 ± 1^a^	1 ± 1^a^
**Anions**						
Chloride (Cl^-^)	49 ± 21^a,b^	39 ± 20^a,b,c^	31 ± 18^b,c,d^	57 ± 14^a^	20 ± 11^c,d^	17 ± 17^d^
Phosphate (PO_4_^3-^)	37 ± 20^a^	32 ± 18^a^	44 ± 14^a,b^	14 ± 10^d^	59 ± 25^b^	75 ± 20^c^
Sulfate (SO_4_^2-^)	10 ± 5^a,b^	25 ± 12^c,d^	23 ± 7^c,d^	25 ± 7^d^	20 ± 15^b,c^	6 ± 5^a^
Nitrate (NO_3_^-^)	4 ± 5^a^	4 ± 3^a^	2 ± 2^a^	4 ± 9^a^	0 ± 1^a^	2 ± 4^a^

All values are mean proportions (%) of n = 15 independent measurements ± SD.

Different letters represent significant differences between the inorganic ion proportion in honeydew of the different hemipteran species.

### Comparison of honeydew and phloem exudates

Phloem exudates of bark tissues from *A*. *alba* and *P*. *abies* were dominated by sucrose (about 60%), but also contained larger proportions of glucose and fructose (about 40%) (S1 Table). No other di- or trisaccharides were detected. In contrast, different proportions of further disaccharides in addition to sucrose as well as different proportions of trisaccharides (mainly melezitose and erlose) were found in all honeydew samples (Table 1, Fig 1).

**Fig 2:**
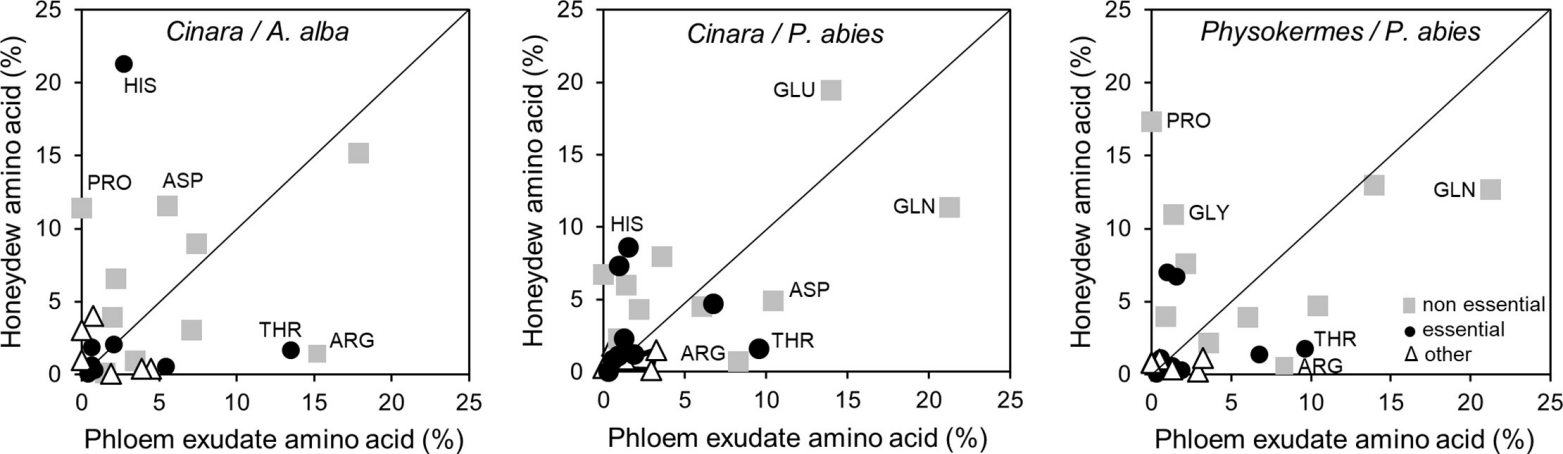
Amino acids in phloem exudate and honeydew, each expressed as percentage of the total amino acid concentration. *Cinara* species feeding on *A*. *alba*, *Cinara* species feeding on *P*. *abies*, and *Physokermes* species feeding on *P*. *abies*. The data show means across both species of a hemipteran genus feeding on the same tree species for the proportion of amino acids in honeydew (data from Table 2). Grey squares: non-essential amino acids, black circles: essential amino acids, white triangles: other organic amino compounds. Points with the highest orthogonal distance to the bisecting line were noted.

All proteinogenic amino acids and some further amino compounds were detected in phloem exudates and in honeydew (Fig 2, S2 Table). GLN and GLU were predominant amino acids in the honeydew of all six hemipteran species (Table 2) and also in the phloem exudates of *A*. *alba* and *P*. *abies* (GLN about 18–19% and GLU 8–15%). In honeydew, there was additionally a considerable amount of PRO and HIS, the proportions of which were much lower in phloem exudates. On the other hand, the proportions of ARG and THR were much higher in the phloem exudate of both tree species (ARG 9–15%; THR 10–13%) than in the honeydew of all hemipteran species (ARG and THR each 1–2%; Table 2).

**Fig 3:**
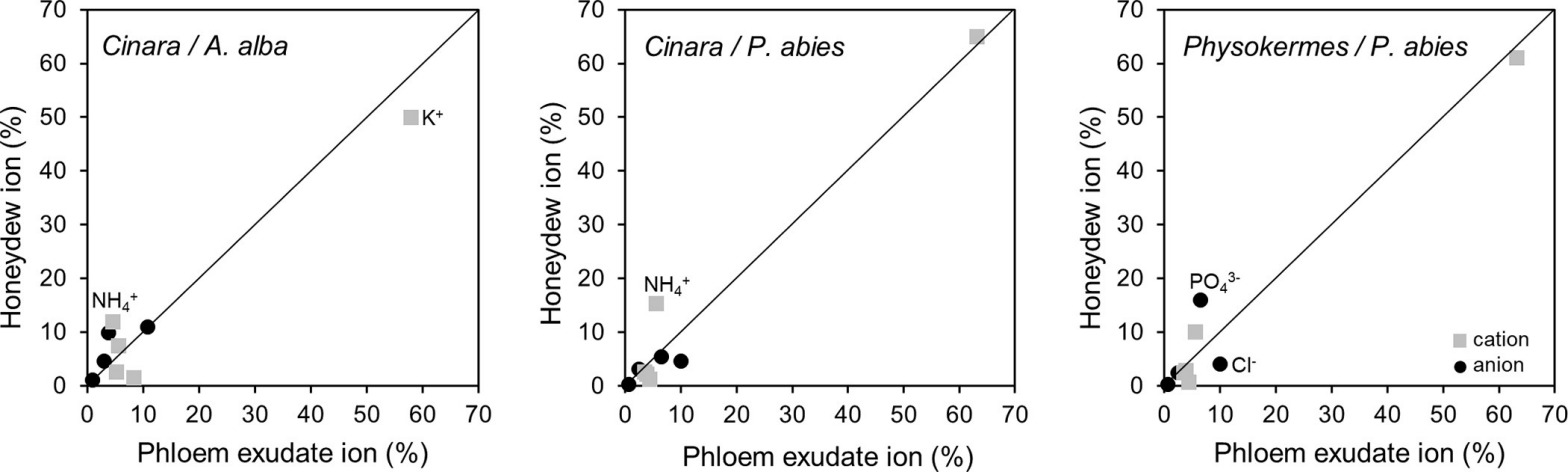
Inorganic ions in phloem exudate and honeydew, each expressed as percentage of the total inorganic ion concentration. *Cinara* species feeding on *A*. *alba*, *Cinara* species feeding on *P*. *abies*, and *Physokermes* species feeding on *P*. *abies*. The data show means across both species of a hemipteran genus feeding on the same tree species for the proportion of inorganic ions in honeydew (data from Table 3). Points with the highest orthogonal distance to the bisecting line were noted. Grey squares: cations, black circles: anions.

Potassium was the most abundant inorganic ion in all honeydew samples and also in the phloem exudate (Fig 3, S3 Table). In honeydew, the most abundant anion was either chloride or phosphate (Table 3), whereas in phloem exudate, it was always chloride.

Honeydew and phloem exudate also showed different ratios sum-of-sugars to sum-of-amino-acids and sum-of-sugars to sum-of-inorganic-anions (Table 4). The ratios were calculated from the total concentration of sugars, amino acids, or inorganic ions either in the honeydew of the different hemipteran species or in the phloem exudate of *A*. *alba* and *P*. *abies*, The ratio sum-of-sugars to sum-of-amino-acids was about 6–7 in phloem exudates of both plant species (Table 4). The corresponding ratio in the honeydew of the different hemipteran species was much higher (2.000–20.000, Table 4). This means that honeydew contains much more sugars in relation to amino acids than phloem exudate. The same applies to the inorganic ions. The ratio sum-of-sugars to sum-of-inorganic-cations was 30–90 and the ratio sum-of-sugars to sum-of-inorganic-anions was 90–900. In phloem exudate, they were about 1–2 and 6–9, respectively (Table 4).

**Table 4:** Ratios of different compounds in phloem exudates of *Abies alba* and *Picea abies* and in the honeydew of different hemipteran species feeding on *A*. *alba* and *P*. *abies*.

	Sugars/amino acids	Sugars/cations	Sugars/anions
***Abies alba***			
Phloem exudate	7 ± 1	2 ± 1	9 ± 3
*C*. *pectinatae* honeydew	4580 ± 3959^a^	68 ± 45^b,c^	274 ± 162^a^
*C*. *confinis* honeydew	2163 ± 2999^a^	55 ± 15^a,b,c^	139 ± 146^a^
***Picea abies***			
Phloem exudate	6 ± 2	1 ± 0	6 ± 2
*C*. *piceae* honeydew	19416 ± 32641^a,b^	90 ± 50^c^	919 ± 713^b^
*C*. *pilicornis* honeydew	3503 ± 3765^a^	69 ± 44^c^	319 ± 137^a^
*P*. *piceae* honeydew	7152 ± 7151^a,b^	28 ± 12^a^	175 ± 95^a^
*P*. *hemicryphus* honeydew	24107 ± 28870^b^	33 ± 24^a,b^	94 ± 90^a^

Values for the ratios in honeydew are means of n = 15 independent measurements ± SD. Values for the ratios in phloem exudate are means of n = 6 independent measurements ± SD. Data are calculated from the concentrations of sugars, amino acids, inorganic cations and anions in phloem exudates and honeydew.

Different letters represent significant differences in sugar-to-amino acids, sugar-to-anions, and sugar-to-cations ratios in honeydew of the different hemipteran species.

### Honeydew composition in relation to hemipteran species and tree species

**Fig 4:**
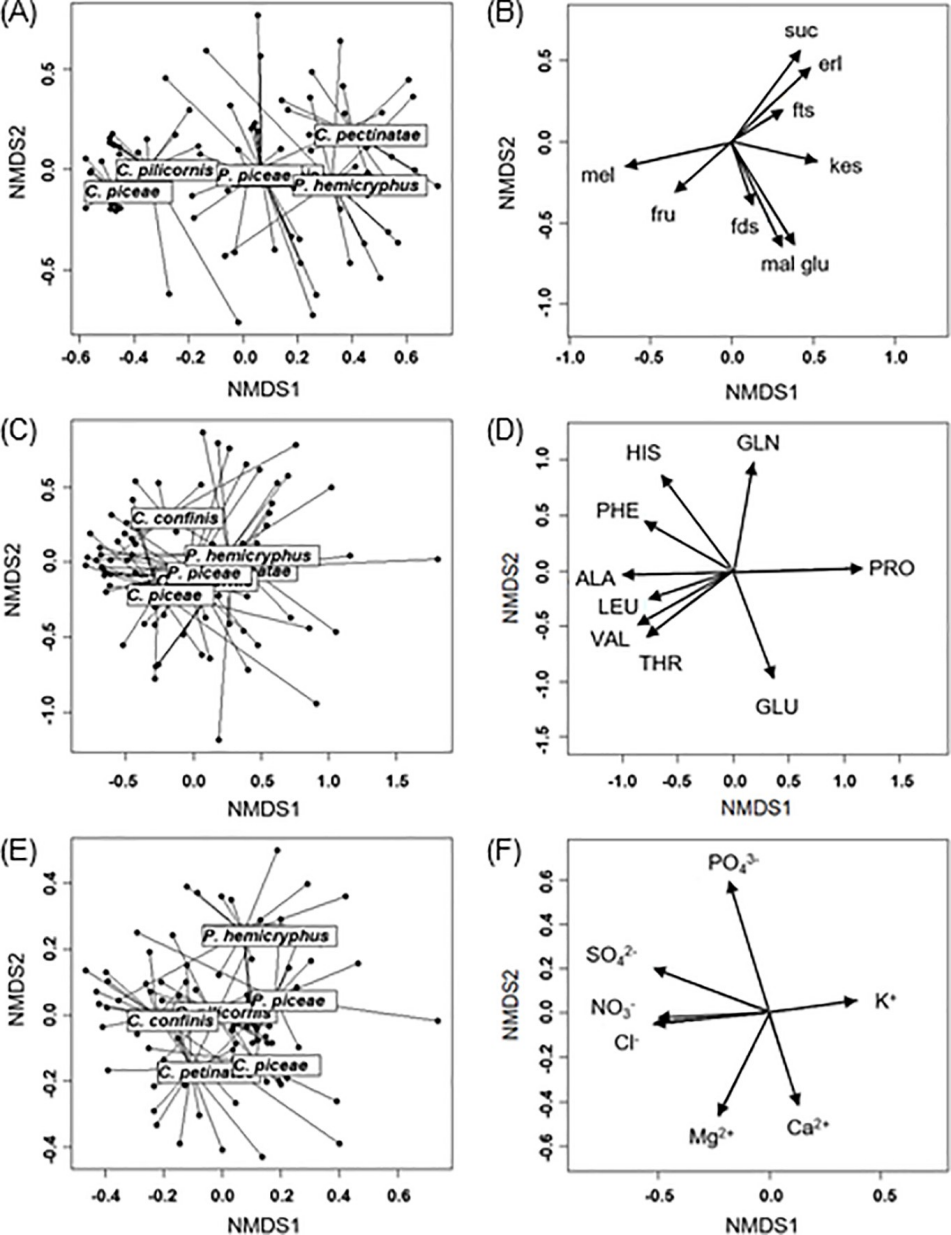
Scatterplots and loadings of NMDS. (A,C,E) Scatterplots of NMCS for (A) sugars, (C) amino acids, and (E) inorganic ions (stress values A = 0.18 C = 0.19, E = 0.19). Samples of each of the hemipteran species are connected with the centroids of the corresponding convex hulls using the function ‘ordispider’ (package Vegan). (B,D,F) Loading plots, which illustrate the original variables (B sugars, D amino acids, F inorganic ions) loaded as vectors in NMDS space. The analyses are based on the proportions of sugars, amino acids, and inorganic ions in honeydew.

In order to reduce the amount and complexity of the data, several NMDS analyses were performed. The scatterplot of analyzed sugars is shown in Fig 4A. There is a visual separation between the samples of *C*. *piceae* and *C*. *pilicornis*, and other hemipteran species samples as well as a visual separation between *C*. *pectinatae* and other hemipteran species.

Fig 4B shows the loading plot of the analyzed sugars in honeydew. The different sugars contribute differently to the separation of the samples. Melezitose is a major contributor to the separation of *C*. *piceae* and *C*. *pilicornis*, whereas sucrose and erlose are major contributors to the separation of *C*. *pectinatae*.

The scatterplot of the amino acids is shown in Fig 4C. In this case, no separation of the hemipteran species was found. The corresponding loading plot of the analyzed amino acids in honeydew is shown in Fig 4D.

The scatterplot of inorganic ions is shown in Fig 4E. There is a visual separation between *P*. *hemicryphus* and the other hemipteran species (Fig 4E). The loading plot of the inorganic ions shows that phosphate is a major contributor to the separation of *P*. *hemicryphus* (Fig 4F).

To support the graphical evaluation, a PERMANOVA and PERMDISP was performed with the same honeydew data using hemipteran species and tree species as categorical variables (Table 5). When considering sugars, there is a high significance for the category of hemipteran species (*p* < 0.001) with 47.8% of the data variation being explained by the hemipteran species and only 13.69% of the data variation being explained by the tree species (*p* < 0.001). In addition, the non-significant values of PERMDISP for sugars indicate that the separations of hemipteran species and tree species in PERMANOVA is caused only by location and not by different dispersion. Considering the amino acids, only 10.7% of the variance is explained by the hemipteran species (*p* < 0.001) and 4.12% by tree species (*p* < 0.001). For inorganic ions only 21.76% of the data variance is influenced by the hemipteran species and 8.85% by tree species (*p* < 0.001). However, the significant values of ions and amino acids in PERMDISP test indicate that there were effects of different dispersions of hemipteran species separation and tree species separation in PERMANOVA. In other words, the significant values in PERMDISP assume that there is an unbalanced group paired with heterogeneity of variance, which makes PERMANOVA very sensitive and promotes type I errors [45].

**Table 5:** Results of multivariate statistical tests PERMANOVA/PERMDISP based on the Euclidean distance matrix of proportions of metabolites and ions in honeydew samples.

	PERMANOVA		PERMDISP	
	Pseudo-F	*p*-value	F	*p*-value
**Hemipteran species**				
Sugars	47.8	0.001	1.65	0.15
Amino acids	10.7	0.001	4.86	>0.001
Ions	21.76	0.001	4.05	0.002
**Tree species**				
Sugars	13.69	0.001	0.285	0.59
Amino acids	4.12	0.001	21.87	>0.001
Ions	8.85	0.001	7.59	0.007

Hemipteran species: *N* (permutations) = 999; *df* = 5

Tree Species: *N* (permutations) = 999; *df* = 1

### Oligosaccharide formation in aphid whole-body homogenates

**Fig 5:**
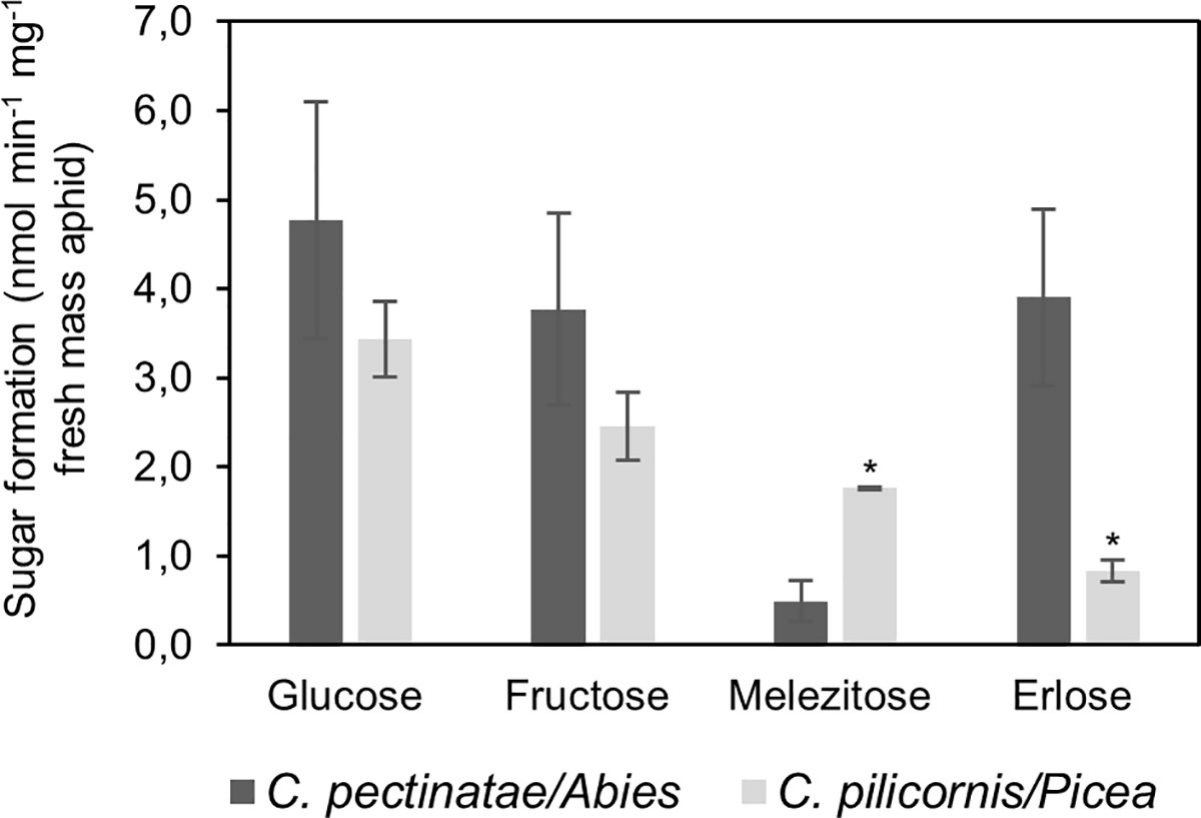
Sugar formation in whole-body homogenates of *C*. *pectinatae* and *C*. *pilicornis*. The aphid homogenates were incubated with 10% sucrose solution. All values are means of n = 3 independent measurements ± SD. Mann-Whitney U tests comparing means of each sugar production rate between different *Cinara* species (* *p* < 0.05) were performed.

Sucrose was converted to monosaccharides and oligosaccharides in the whole-body homogenates of both *Cinara* species (Fig 5). In the extracts of *C*. *pectinatae*, more erlose than melezitose was produced, whereas in the extracts of *C*. *pilicornis*, more melezitose than erlose was produced.

## Discussion

Honeydew of hemipteran species feeding on conifers like spruce or fir is often the basis for honeydew honey; therefore, the quality of it is also influenced by the chemical composition of the honeydew [30]. For beekeepers and for the honey industry it is important to know which factors influence the chemical composition of honeydew, be it the hemipteran species, the host trees, and/or environmental factors.

### Origin of sugars in honeydew

Phloem exudates of bark tissues from *A*. *alba* and *P*. *abies* contained sucrose, glucose and fructose. In contrast, pure phloem sap normally does not contain glucose and fructose [2]. Ziegler and Mittler [46] collected phloem sap from *P*. *abies* with the help of aphid stylectomy and found that sucrose was the only sugar in the phloem. Hexoses in the phloem exudate stem mainly from artificially hydrolyzed sucrose by the activity of sucrose cleavage enzymes from the wounded surface of the bark [2]. This may also be assumed for *A*. *alba*, because the bark exudate also contained sucrose and glucose and fructose, but no other di- or trisaccharides.

In contrast, honeydew of the different hemipteran species feeding on fir or spruce contained hexoses like glucose and fructose as well as a wide variety of oligosaccharides which do not typically occur in the phloem sap (Table 1). This corresponds to the results of Mittler [21], who showed that glucose, fructose, sucrose, and melezitose were present in the honeydew of the aphids *Tuberolachnus salignus* feeding on willow, but not in the phloem sap of willow, where only sucrose was present. Therefore, sucrose ingested by the phloem-feeding insects is hydrolyzed into glucose and fructose in the insects’ digestive tract. Fisher et al. [22] suggested that, after hydrolyzation of sucrose, a large portion of the glucose is transformed by the aphid into glucose-containing oligomers for osmoregulation of ingested phloem sap. The observed predominance of fructose over glucose and the presence of large quantities of glucose-containing oligosaccharides in honeydew (Table 1) supports that. High sucrase activity, an α-glucosidase which probably also shows transglucosidation activity, was found in pea aphids [25,26,47]. Furthermore, crushed aphids incubated with sucrose solution produced glucose, fructose, melezitose, erlose, and other oligosaccharides [19], with melezitose being a biosynthetic end product in aphid carbohydrate metabolism [25]. The enzymatic hydrolysis and oligosaccharide-forming transglycosidation reactions show considerable specificity for sucrose, whereas other sugars remain unaffected [25]. However, the enzymatic level of transformations of dietary sucrose or other sugars in the insect gut is not yet completely understood [1].

### Origin of amino acids in honeydew

The ratios sugar-to-amino-acids were much lower in the phloem exudate of the host plants than in the honeydew of the different hemipteran species (Table 4). Hemipterans are very efficient in absorbing amino acids from the ingested phloem sap, as only 1–3% of the amount ingested was found in honeydew of different aphid species [9].

Weibull et al. [48] showed that the amino acid composition of phloem samples taken from leaves via excised aphid stylets and that of exudates from cut leaves were highly correlated. Thus, the exudate technique offers a proper alternative to the aphid stylet technique for studying the composition of phloem sap [48]. Although all proteinogenic amino acids were found in phloem exudates and honeydew, the composition was different (Fig 2). Most essential amino acids were found in lower proportions in honeydew compared to phloem exudate; the proportion of HIS, however, was increased. This may be an indication for an active regulation mechanism in the insects and varying uptakes of individual amino acids, probably as a result of variable supply and demand [11].

In general, the main nitrogen source of phloem-feeding hemipterans are free amino acids of the phloem sap [1]. In addition, these insects also produce amino acids from sucrose carbon [11] or from the carbon of dietary amino acids [49]. Symbiotic microorganisms, several species of *Buchnera*, are known to be involved in the synthesis of amino acids, including some essential amino acids [1,50].

### Origin of inorganic ions in honeydew

The composition of inorganic ions in honeydew of the different hemipteran species roughly reflect that composition in the phloem exudate of *A*. *alba* and *P*. *abies*. The main cation in the honeydew of all analyzed hemipteran species was potassium (50–80% of the total cation content), whereas the proportion of sodium was much lower (Table 3). Similar ratios of potassium-to-sodium were found in the honeydew of *Megoura viciae* feeding on *Vicia faba* [51] or in the honeydew of *Myzus persicae* feeding on *Aster tripolium* [52]. This is probably caused by the diet of the insects. It was proposed that there is also a correlation between the diet of an insect and the ratio of potassium-to-sodium in haemolymph: in carnivorous insects, this ratio is lower and in herbivorous insects it is higher [53].

Honeydew honey contains more inorganic cations and anions than blossom (nectar) honey [54]. It could be assumed that this difference is caused by different ratios of sugars-to-inorganic ions in honeydew and floral nectar, assuming that they are lower in nectar. However, the sugar-to-cation and the sugar-to-anion ratios (Table 4) were similar or even higher in honeydew than in floral nectar of other plant species [38,55]. Therefore, differences in the content of inorganic ions in both types of honey must have further and other reasons.

### Sugar composition is influenced by the hemipteran species

The analysis of the data shows that the hemipteran species has a much higher influence on the sugar composition of the honeydew than the tree species (Table 5). In the case of the amino acids or ions, the variance of the data cannot be elucidated by either the hemipteran species or the conifer species. This raises the question whether there are models or selective agents beyond this to predict the honeydew composition. The honeydew composition may also depend on several environmental factors, like weather conditions or ant tending as well as the developmental stage of the host plant or the insect [9,18].

It has been shown that melezitose has negative effects on the quality of honeydew honey, and the so-called “cement honey” is associated with the melezitose content [56]. Furthermore, melezitose can have negative effects on overwintering honey bees [57]. The results of the present study show that the sucking hemipteran and not the conifer species is primarily responsible for the diversity of the oligosaccharides, especially melezitose and erlose, in honeydew (Table 5). This corresponds with the results of other hemipteran and host plant species. Bacon and Dickinson [19] found that the honeydew of the aphid *Eucallipterus tiliae* (L.) feeding on lime tree contained appreciable amounts of melezitose, whereas in the honeydew of the scale insect *Eulecanium coryli* (L.) feeding also on lime tree, no melezitose could be detected. A wide variation in the proportions of erlose and melezitose in honeydew was also observed for eight aphid species feeding on *Tanacetum vulgare* [4].

Moreover, incubation of sucrose with whole-body homogenates of *C*. *pectinatae* and *C*. *pilicornis* resulted in similar oligosaccharide patterns as were found in honeydew samples (Fig 5). This indicates that different aphid species probably have different enzymatic activities, which lead to different oligosaccharide compositions in honeydew. The total rate of sucrose hydrolyses was similar in both aphid species (about 1.5 mg sucrose mg^-1^ fresh weight of aphid day^-1^) and with that corresponds to the rates of sucrose hydrolyses reported by Bacon and Dickinson ([19]; about 1 mg sucrose mg^-1^ fresh weight of aphid day^-1^).

Liebig [20] found higher proportions of melezitose (up to 20%) in the honeydew of *C*. *pectinatae* on *A*. *alba* than was shown in this study (2%). Reasons for this difference may be the high variability between samples, even if taken from the same hemipteran species and the same host plant [18], or different environmental conditions during sample collection. Unfortunately, in the study by Liebig [20], the proportion of the other important trisaccharide, erlose, was not shown. Therefore, it cannot be excluded that the two trisaccharides were not separated.

The proportion of melezitose in honeydew was related to the presence or absence of ants [18]. Woodring et al. [4] proposed that ants are attracted to melezitose not because it is itself a valuable food source, but because it is associated with a very sugar-rich honeydew. In the present study, ant-tending was not analyzed in detail. Differences in ant-tending among the *Cinara* species are known from the literature; *C*. *piceae* (on spruce) and *C*. *confinis* (on fir) are more often tended by ants while *C*. *pilicornis* (on spruce) and *C*. *pectinatae* (on fir) are rarely tended by ants [30]. Considering these general data, there is no correlation between ant-tending and melezitose content in honeydew of the analyzed *Cinara* species. However, the variation in honeydew composition within the hemipteran species may in part reflect variation in ant-hemipteran interaction.

## Conclusion

In conclusion, the sucking hemipteran and not the host plant is primarily responsible for the diversity of the sugars, especially the oligosaccharides in honeydew. In the case of inorganic ions, mainly the proportions of chloride, phosphate, and ammonium showed significant differences between the honeydews of different hemipteran species. In contrast, the composition of amino acids in honeydew was rather similar across the hemipteran species.

## Supporting information

S1 Table Sugar composition in phloem exudates of *Abies alba* and *Picea abies*. All values are mean proportions (%) of n = 6 independent measurements ± SD.
(PDF)

S2 Table Amino acid composition in phloem exudates of *Abies alba* and *Picea abies*. All values are mean proportions (%) of n = 6 independent measurements ± SD.
(PDF)

S3 Table Inorganic cation and anion composition in phloem exudates of *Abies alba* and *Picea abies*. All values are mean proportions (%) of n = 6 independent measurements ± SD.
(PDF)

## References

[1] Douglas AE. Phloem-sap feeding by animals: problems and solutions. J Exp Bot. 2006; 57: 747–754. 10.1093/jxb/erj067 16449374

[2] Fink D, Dobbelstein E, Barbian A, Lohaus G. Ratio of sugar concentrations in the phloem sap and the cytosol of mesophyll cells in different tree species as an indicator of the phloem loading mechanism. Planta. 2018; 248: 661–673. 10.1007/s00425-018-2933-7 29882156

[3] Lohaus G, Moellers C. Phloem transport of amino acids in two *Brassica napus* L. genotypes and one *B*. *carinata* genotype in relation to their seed protein content. Planta. 2000; 211: 833–840. 10.1007/s004250000349 11144268

[4] Woodring J, Wiedemann R, Fischer M K, Hoffmann K H, Völkl W. Honeydew amino acids in relation to sugars and their role in the establishment of ant-attendance hierarchy in eight species of aphids feeding on tansy (*Tanacetum vulgare*). Physiol Entomol. 2004; 29: 311–319.

[5] Nadwodnik J, Lohaus G. Subcellular concentrations of sugar alcohols and sugars in relation to phloem translocation in *Plantago major*, *Plantago maritima*, *Prunus persica*, and *Apium graveolens*. Planta. 2008; 227: 1079–1089. 10.1007/s00425-007-0682-0 18188589 PMC2270920

[6] Öner-Sieben S, Lohaus G. Apoplastic and symplastic phloem loading in *Quercus robur* and *Fraxinus excelsior*. J Exp Bot. 2014; 65: 1905–1916. 10.1093/jxb/eru066 24591056 PMC3978624

[7] Karley AJ, Douglas AE, Parker WE. Amino acid composition and nutritional quality of potato leaf phloem sap for aphids. J Exp Biol. 2002; 205: 3009–3018. 12200404 10.1242/jeb.205.19.3009

[8] Lohaus G, Schwerdtfeger M. Comparison of sugars, iridoid glycosides and amino acids in nectar and phloem sap of *Maurandya barclayana*, *Lophospermum erubescens*, and *Brassica napus*. PLoS ONE. 2014; 9(1): e87689 10.1371/journal.pone.0087689 24489951 PMC3906183

[9] Sandström JP, Moran NA. Amino acid budgets in three aphid species using the same host plant. Physiol Entomol. 2001; 26: 202–211.

[10] Wilkinson TL, Ashford DA, Pritchard J, Douglas AE. Honeydew sugars and osmoregulation in the pea aphid *Acyrthosiphon pisum*. J Exp Biol 1997; 200: 2137–2143. 9320049 10.1242/jeb.200.15.2137

[11] Febvay G, Rahbé Y, Rynkiewicz M, Guillaud J, Bonnot G. Fate of dietary sucrose and neosynthesis of amino acids in the pea aphid, *Acyrthosiphon pisum*, reared on different diets. J Exp Biol. 1999; 202: 2639–2652. 10482723 10.1242/jeb.202.19.2639

[12] Auclair J. Aphid feeding and nutrition. Ann Rev Entomol. 1963; 8: 439–490.

[13] Leroy P, Wathelet B, Sabri A, Francis F, Verheggen F, Capella Q, et al Aphid-host plant interactions: does aphid honeydew exactly reflect the host plant amino acid composition? Arthropod-Plant Interact. 2011; 5: 193–199.

[14] Sabri A, Vandermoten S, Leroy PD, Haubruge E, Hance T, Thonart P, et al Proteomic investigation of aphid honeydew reveals an unexpected diversity of proteins. PloS ONE. 2013; 8: e74656 10.1371/journal.pone.0074656 24086359 PMC3783439

[15] Völkl W, Woodring J, Fischer M, Lorenz MW, Hoffmann KH. Ant-aphid mutualisms: the impact of honeydew production and honeydew sugar composition on ant preferences. Oecologia. 1999; 118: 483–491. 10.1007/s004420050751 28307416

[16] Fischer MK, Völkl W, Schopf R, Hoffmann KH. Age-specific pattern in honeydew production and honeydew composition in the aphid *Metopeurum fuscoviride*: implications for ant-attendance. J Insect Physiol. 2002; 48: 319–326. 10.1016/s0022-1910(01)00179-2 12770106

[17] Hendrix DL, Wei Y, Legget JE. Homopteran honeydew is determined by both the insect and the plant species. Comp Biochem Physiol. 1992; 101: 23–27.

[18] Fischer MK, Shingleton AW. Host plant and ants influence the honeydew sugar composition of aphids. Funct Ecol. 2001; 15: 544–550.

[19] Bacon JSD, Dickinson B. The origin of melezitose: a biochemical relationship between the lime tree (*Tilia* spp.) and an aphis (*Eucallipterus tiliae* L.). Biochem. 1957; 66: 289–297. 10.1042/bj0660289 PMC1200007 13445686

[20] Liebig G. Gaschromatographische und enzymatische Untersuchungen des Zuckerspektrums des Honigtaus von *Buchneria pectinatae* (Nördl.) Apidologie. 1979; 10: 213–225.

[21] Mittler TE. Studies on the feeding and nutrition of *Tuberolachnus salignus* (Gmelin) (Homoptera, Aphididae). II. The nitrogen and sugar composition of ingested phloem sap and excreted honeydew. J Exp Biol. 1958; 35: 74–84.

[22] Fisher DB, Wright JP, Mittler TE. Osmoregulation by the aphid *Myzus persicae*: a physiological role for honeydew oligosaccharides. J Insect Physiol. 1984; 30: 387–393.

[23] Rhodes JD, Croghan PC, Dixon AFG. Dietary sucrose and oligosaccharide synthesis in relation to osmoregulation in the pea aphid, *Acythosiphon pisum*. Physiol Entomol. 1997; 22: 373–379.

[24] Ashford DA, Smith WA, Douglas AE. Living on a high sugar diet: the fate of sucrose ingested by a phloem feeding insect, the pea aphid *Acyrthosiphon pisum*. J Insect Physiol. 2000; 46: 335–341. 10.1016/s0022-1910(99)00186-9 12770238

[25] Walters FS, Mullin CA. Sucrose-dependent increase in the oligosaccharide production and associated glycosidase activities in the potato aphid *Macrosiphum euphorbiae* (Thomas). Arch Insect Biochem Physiol. 1988; 9: 35–46.

[26] Braendle C, Miura T, Bickel R, Shingleton AW, Kamphampati S, Stern DL. Developmental origin and evolution of bacteriocytes in the aphid-*Buchnera* symbiosis. PLoS Biol. 2003; 1: e1 10.1371/journal.pbio.0000001 14551917 PMC212699

[27] Prosser WA, Douglas AE. A test of the hypothesis that nitrogen is upgraded and recycled in an aphid (*Acythosiphon pisum*) symbiosis. J Insect Physiol. 1992; 38: 93–99.

[28] Sasaki T, Ishikawa H. Production of essential amino acids from glutamate by mycetocyle symbionts of the pea aphid, *Acythosiphon pisum* maintained on artificial diets. J Insect Physiol. 1995; 37: 749–756.

[29] Wilkinson TL, Adams D, Minto LB, Douglas AE. The impact of host plant on the abundance and function of symbiotic bacteria in an aphid. J Exp Biol. 2001; 204: 3027–3028. 11551991 10.1242/jeb.204.17.3027

[30] Kunkel H, Kloft, Die Honigtau-Erzeuger des Waldes W. J., in: Kloft W.J. and Kunkel H. (Eds.), Waldtracht und Waldhonig in der Imkerei. Ehrenwirth, Munich 1985; pp. 48–265.

[31] Ruoff K, Luginbühl W, Kilchenmann V, Bosset JO., von der Ohe K, von der Ohe W, et al Authentication of the botanical origin of honey using profiles of classical measurands and discriminant analysis. Apidologie. 2007; 38: 438–452.

[32] Nottbohm FE, Lucius F. Melezitose im Honigtauhonig der Linde. Zeitschrift für Untersuchungen der Lebensmittel. 1929; 57: 549–558.

[33] Hudson CS, Sherwood SF. The occurrence of melezitose in honey. J Am Chem Soc. 1920; 42: 116–125.

[34] Gölz H. Der Melezitosegehalt im Honigtau verschiedener Lachnidenarten. Apidologie. 1982; 13: 89–91.

[35] Zoebelein G. Die Rolle des Waldhonigtaus im Nahrungshaushalt forstlich nützlicher Insekten. Forstw Centralbl. 1957; 76: 24–34.

[36] Hijaz F, Killiny N. Collection and chemical composition of phloem sap from *Citrus sinensis* L. Osbeck (Sweet Orange). PLoS ONE. 2014 9(7): e101830 10.1371/journal.pone.0101830 25014027 PMC4094394

[37] Findling S, Zanger K, Krueger S, Lohaus G. Subcellular distribution of raffinose oligosaccharides and other metabolites in summer and winter leaves of *Ajuga reptans* (Lamiaceae). Planta. 2015; 241: 229–241. 10.1007/s00425-014-2183-2 25269399

[38] Göttlinger T, Schwerdtfeger M, Tiedge K, Lohaus G. What do nectarivorous bats like? Nectar composition in Bromeliaceae with special emphasis on bat-pollinated species. Front Plant Sci. 2019; 10: 205 10.3389/fpls.2019.00205 30847001 PMC6393375

[39] West SG, Finch JF, Curran PJ. Structural equation models with nonnormal variables: problems and remedies In: Hoyle RH, editor. Structural equation modeling: Concepts, issues and applications. Newbery Park, CA: Sage; 1995 p56–75.

[40] Hervé MR, Nicolè F, Lê Cao KA. Multivariate analysis of multiple datasets: a practical guide for chemical ecology. J Chem Ecol. 2018; 44: 215–234. 10.1007/s10886-018-0932-6 29479643

[41] Ramette A. Multivariate analyses in microbial ecology. FEMS Microbiol Ecol. 2007; 62: 142–160. 10.1111/j.1574-6941.2007.00375.x 17892477 PMC2121141

[42] Oksanen J, Kindt R, Legendre P, O’Hara B, Henry M, Stevens H. The vegan package. Community ecology package. R package version 2.5–3 [online]. 2007; https://CRAN.R-project.org/package=vegan (accessed on 07 October 2019).

[43] Anderson MJ. A new method for non-parametric multivariate analysis of variance. Austral Ecology. 2001; 26: 32–46.

[44] Anderson MJ. Distance-based tests for homogeneity of multivariate dispersions. Biometrics. 2006; 62: 245–253. 10.1111/j.1541-0420.2005.00440.x 16542252

[45] Anderson MJ, Walsh DCI. PERMANOVA, ANOSIM, and the Mantel test in the face of heterogeneous dispersions: what null hypothesis are you testing? Ecol Monogr. 2013; 83: 557 574.

[46] Ziegler H, Mittler TE. Über den Zuckergehalt der Siebröhren- bzw- Siebzellensäfte von *Heracleum mantegazzianum* und *Picea abies* (L.) Karst Z Naturforschung. 1959; 14 B: 278–281.

[47] Cristofoletti PT, Ribeiro AF, Deraison C, Rahbe Y, Terra WR. Midgut adaption and digestive enzyme distribution in a phloem feeding insect, the pea aphid *Acyrthosiphon pisum*. J Insect Physiol 2003; 49: 11–24. 10.1016/s0022-1910(02)00222-6 12770012

[48] Weibull J, Ronquist F, Brishammar, S. Free amino acid composition of leaf exudates and phloem sap. Plant Physiol. 1990; 92: 222–226. 10.1104/pp.92.1.222 16667250 PMC1062273

[49] Haribal M, Jander G. Stable isotope studies reveal pathways for the incorporation of non-essential amino acids in *Acyrthosiphon pisum* (pea aphids). J Exp Biol. 2015; 218: 3797–3806. 10.1242/jeb.129189 26632455

[50] Sasaki T, Fukuchi N, Ishikawa H. Amino acid flow through aphid and its symbiont: studies with ^15^N-labeled glutamine. Zool Sci. 1993; 10: 787–791.

[51] Ehrhardt P. Die anorganischen Bestandteile des Honigtaues von *Megoura viciae* Buckt. Experientia. 1965; XXI/8: 472–473.

[52] Downing N. The regulation of sodium, potassium and chloride in an aphid subjected to ionic stress. J Exp Biol. 1980; 87: 343–349.

[53] Wyatt GR. The biochemistry of insect haemolymph. Ann Review Entomol. 1961; 6: 75–102.

[54] Fermo P, Beretta G, Facino RM, Gelmini F. Ionic profile of honey as a potential indicator of botanical origin and global environmental pollution. Environ Pollution. 2013; 178: 173–181. 10.1016/j.envpol.2013.03.029 23583673

[55] Tiedge K, Lohaus G. Nectar sugars and amino acids in day- and night-flowering Nicotiana species are more strongly shaped by pollinators’ preferences than organic acids and inorganic ions. PLoS ONE. 2017; 12(5): e0176865 10.1371/journal.pone.0176865 28467507 PMC5415175

[56] Schmelz H, Gall H, Fehlinger GF, Heider E, Rösch J. Alle Jahre wieder Ratlosigkeit–oder was tun bei Melezitosetracht. ADIZ. 2002; 1: 23–24

[57] Imdorf A, Bogdanov S, Kilchenmann V. Zementhonig im Honig- und Brutraum–was dann? Schweizer Zentrum für Bienenforschung. 2002; 1: 1–16.

