# Supplementary material for: Sugar, amino acid and inorganic ion profiling of the honeydew from different hemipteran species feeding on Abies alba and Picea abies
Source: PLoS One. 2020 Jan 24;15(1):e0228171. doi: 10.1371/journal.pone.0228171 (PMC6980476; doi:10.1371/journal.pone.0228171)
Supplement: S1 Table — All values are mean proportions (%) of n = 6 independent measurements ± SD. (PDF) [file pone.0228171.s001.pdf]

S1 Table. Sugar composition in phloem exudates of *Abies alba* and *Picea abies*. All values are mean proportions (%) of n = 6 independent measurements ± SD.

**Sugar [%]**	***Abies alba***	***Picea abies***
Glucose (glu)	19 ± 4	15 ± 2
Fructose (fru)	24 ± 4	27 ± 2
Sucrose (suc)	57 ± 8	58 ± 3
