# Supplementary material for: Sugar, amino acid and inorganic ion profiling of the honeydew from different hemipteran species feeding on Abies alba and Picea abies
Source: PLoS One. 2020 Jan 24;15(1):e0228171. doi: 10.1371/journal.pone.0228171 (PMC6980476; doi:10.1371/journal.pone.0228171)
Supplement: S2 Table — All values are mean proportions (%) of n = 6 independent measurements ± SD. (PDF) [file pone.0228171.s002.pdf]

**S2 Table. Amino acid composition in phloem exudates of *Abies alba* and *Picea abies***. All values are mean proportions (%) of n = 6 independent measurements ± SD.

| **Amino acid [%]** | ***Abies alba*** | ***Picea abies*** |
|---|---|---|
| Glutamate (GLU) | 7.6 ± 1.2 | 15.0 ± 2.3 |
| Glutamine (GLN) | 18.1 ± 4.6 | 19.4 ± 9.4 |
| Aspartate (ASP) | 5.6 ± 0.8 | 11.7 ± 3.7 |
| Asparagine (ASN) | 2.2 ± 0.7 | 1.9 ± 1.5 |
| Proline (PRO) | 0.1 ± 0.0 | 0.1 ± 0.1 |
| Glycine (GLY) | 2.0 ± 0.6 | 1.5 ± 0.6 |
| Serine (SER) | 7.2 ± 0.9 | 5.9 ± 1.8 |
| Alanine (ALA) | 3.6 ± 2.2 | 3.8 ± 1.1 |
| Tyrosine (TYR) | 1.5 ± 0.2 | 0.8 ± 0.5 |
| Arginine (ARG) | 14.9 ± 3.1 | 8.8 ± 5.2 |
| Histidine (HIS) | 2.6 ± 0.6 | 1.5 ± 0.2 |
| Lysine (LYS) | 2.0 ± 0.2 | 0.9 ± 0.2 |
| Threonine (THR) | 13.2 ± 2.4 | 10.0 ± 5.2 |
| Valine (VAL) | 5.4 ± 0.3 | 6.4 ± 1.7 |
| Isoleucine (ILE) | 0.6 ± 0.1 | 1.5 ± 1.3 |
| Leucine (LEU) | 0.7 ± 0.3 | 1.1 ± 0.7 |
| Phenylalanine (PHE) | 0.6 ± 0.1 | 0.5 ± 0.2 |
| Tryptophan (TRP) | 0.8 ± 0.3 | 0.9 ± 0.4 |
| Methionine (MET) | 0.4 ± 0.2 | 0.2 ± 0.2 |
| Non-proteinogenic amino acids (NP) | 10.9 ± 3.0 | 8.1 ± 2.7 |
