# Supplementary material for: Sugar, amino acid and inorganic ion profiling of the honeydew from different hemipteran species feeding on Abies alba and Picea abies
Source: PLoS One. 2020 Jan 24;15(1):e0228171. doi: 10.1371/journal.pone.0228171 (PMC6980476; doi:10.1371/journal.pone.0228171)
Supplement: S3 Table — All values are mean proportions (%) of n = 6 independent measurements ± SD. (PDF) [file pone.0228171.s003.pdf]

S3 Table. Inorganic cation and anion composition in phloem exudates of *Abies alba* and *Picea abies*. All values are mean proportions (%) of n = 6 independent measurements ± SD.

**Ion [%]**	***Abies alba***	***Picea abies***
**Cations**		
Potassium (K^+)	71.3 ± 3.6	77.4 ± 3.0
Sodium (Na^+)	5.9 ± 3.0	5.3 ± 0.7
Ammonium (NH_4^+)	7.2 ± 3.5	5.8 ± 1.9
Magnesium (Mg^{2+})	5.9 ± 1.5	5.0 ± 1.1
Calcium (Ca^{2+})	9.7 ± 2.6	6.5 ± 2.3
**Anions**		
Chloride (Cl^-)	59.6 ± 10.1	52.7 ± 5.7
Phosphate (PO_4^{3-})	22.1 ± 13.1	29.0 ± 6.7
Sulfate (SO_4^{2-})	13.5 ± 6.0	14.1 ± 2.0
Nitrate (NO_3^-)	4.9 ± 2.3	4.2 ± 2.2
